# Association of meal frequency with metabolic syndrome in Korean adults: from the Korea National Health and Nutrition Examination Survey (KNHANES)

**DOI:** 10.1186/s13098-017-0277-2

**Published:** 2017-10-03

**Authors:** Chan-Hee Jung, Ji Sung Lee, Hee Jung Ahn, Jin-Sun Choi, Min Young Noh, Ji Jeung Lee, Eun Young Lee, Jeong Hyun Lim, Young Ran Lee, So Yoon Yoon, Chong Hwa Kim, Dong-Hyeok Cho, Young Sik Choi, Kyung Mook Choi

**Affiliations:** 1 Division of Endocrinology and Metabolism, Department of Internal Medicine, Soonchunhyang University School of Medicine, Bucheon, South Korea; 2 Clinical Research Center, Asan Medical Center, Seoul, South Korea; 3 Department of Nutrition, Eulji Hospital, Seoul, South Korea; 4 Department of Nutrition, Sungkyunkwan University, Kangbuk Samsung Hospital, Seoul, South Korea; 5 Department of Nutrition, Seoul St. Mary’s Hospital, The Catholic University, Seoul, South Korea; 6 Department of Nutrition, Inje University Sanggye Paik Hospital, Seoul, South Korea; 7 Department of Nutrition Service, Inje University Ilsanpaik Hospital, Ilsan, South Korea; 8 Department of Food Service and Nutrition Care, Seoul National University Hospital, Seoul, South Korea; 9 Department of Nutrition, Dongtan Sacred Heart Hospital, Dongtan, South Korea; 10 Department of Dietetics and Nutrition Service Team, Asan Medical Center, Seoul, South Korea; 11 Department of Internal Medicine, Sejong General Hospital, Bucheon, South Korea; 12 Department of Internal Medicine, Chonnam National University Hospital, Gwangju, South Korea; 13 Departments of Internal Medicine, Kosin University College of Medicine, Busan, South Korea; 14 Division of Endocrinology and Metabolism, Department of Internal Medicine, Korea University Guro Hospital, 80 Guro-Dong, Guro-Gu, Seoul, 152-050 South Korea

**Keywords:** Meal frequency, Metabolic syndrome, Korean National Health and Nutrition Examination Survey

## Abstract

**Background:**

Although previous studies have established a close relationship between caloric intake and metabolic syndrome, there is limited research exploring the impact of meal frequency adjusted by caloric intake on metabolic syndrome (MetS).

**Objective:**

To evaluate the association of meal frequency and MetS after adjusting for confounding factors including caloric intake in Korean men and women.

**Methods:**

We analyzed the national representative data of a total 12,389 adults (5171 men, 7218 women) from the Korean National Health and Nutrition Examination Survey (KNHANES) 2010–2012. Subjects were categorized as eating 3 meals/day (MF3) or 2 or fewer meals/day (MF ≤ 2). Daily caloric intake was calculated using CAN-Pro 4.0 (The Korean Nutrition Society, Seoul, Korea).

**Results:**

The prevalence of components of MetS differed significantly according to meal frequency in both men and women. In an unadjusted analysis, the prevalence of MetS in women was significantly higher in the MF3 group than the MF ≤ 2 group (27.5% vs. 17.8%, *P* < 0.001), whereas the prevalence of MetS in men did not differ between the MF3 and MF ≤ 2 groups (24.6% vs. 22.7%, *P* = 0.281). However, after adjusting for age, caloric intake, smoking status, alcohol consumption, physical activity, income, and education level, men in the MF ≤ 2 group had an increased risk of metabolic syndrome compared to men in the MF3 group (OR = 1.37, 95%, CI = 1.12–1.67). On the other hand, meal frequency did not affect the risk of metabolic syndrome in women after adjusting for confounding factors including caloric intake (OR = 1.09, 95%, CI = 0.90–1.31).

**Conclusions:**

This study suggests that lower meal frequency adjusted for caloric intake, physical activity, age, smoking, alcohol, income, and education may be associated with increased risk of MetS in Korean men.

## Background

Metabolic syndrome (MetS) is a cluster of factors associated with an increased risk of developing cardiovascular disease (CVD) and type 2 diabetes [1]. MetS is known to be a complex interaction among genetic, metabolic, and environmental factors such as physical activity and diet [2]. Over the last several decades, the prevalence of MetS has increased, and it has become a major public health concern worldwide, including in Korea [3]. Identifying ways to reduce the risk of MetS is very important to public health. Among modifiable environmental factors, dietary habits are of central importance in the prevention and treatment of MetS.

Although the modern Westernized lifestyle is characterized by irregular meal pattern such as skipped meals, consumption of 3 meals/day is the most common pattern of eating. Also, there is a long-held belief that three regular meals consumption per day are beneficial to metabolic health and therefore is recommended to the general population, although there is limited scientific evidence to support this recommendation. In addition, research about relationship between dietary pattern and MetS is limited [4–6].

Several epidemiologic and clinical data suggest that regular meal patterns appear beneficial to metabolic profiles. Sierra-Johnson et al. reported that, in a population-based study of 3607 individuals, aged 60 years, eating regular meals was associated with a lower risk of MetS and insulin resistance compared to that in irregular eaters who eat 1 or 2 meals/day [7]. A study of Finnish adolescents found that 5 meals/day had a reduced risk of being overweight/obese in both genders and reduced abdominal obesity in boys compared to 4 or fewer meals/day [8]. Carlson et al. reported that consuming 1 meal/day without calorie restriction during a 2-month period exhibited elevated fasting plasma glucose and impaired glucose tolerance associated with a delayed insulin response compared to those consuming 3 meals/day [9].

On the other hand, other studies suggest that subjects who have reported reduced meal frequency appear beneficial health outcomes [10]. Intermittent fasting has attracted much attention worldwide because it can increase longevity in animals and also has beneficial effects on risk factors for CVD and several other diseases including cancer, diabetes, and neurologic disorders [11–13]. Several studies related to Ramadan fasting as a unique natural model of intermittent fasting, showed improvements in risk profiles of CVD [14]. As a result, reduced meal frequency by intermittent fasting supported an association between lower meal frequency and reduced risk of MetS. Although the question of whether the effects of reduced meal frequency are the result of caloric restriction or fasting itself is not resolved, at least some of the beneficial effects of intermittent fasting are suggested to be independent of calorie intake [15].

Caloric intake has been established as the most fundamental factor for longevity and health outcome. Thus, research exploring the effects of meal frequency should consider caloric intake at the same time because the effect of meal frequency on MetS would be difficult to isolate from any effect induced by calorie restriction. Therefore, we evaluated the association between meal frequency adjusted by caloric intake and MetS using a large nationally representative dataset. We hypothesized that, in the general population, eating 3 meals/day would be associated with a different risk of MetS than eating 2 or fewer less meals/day after adjusting for confounding factors including caloric intake.

## Methods

### Study design and subjects

The datasets used in this study originated from the fifth KNHANES (2010–2012), which is a national survey program that has been assessing the health and nutritional status of Koreans since 1998. The survey combines health and dietary interviews with standardized physical examinations and laboratory tests to provide a broad perspective on health risk behaviors and indicators, and chronic diseases. A total of 21,595 individuals took part in the KNHANES 2010–2012 (2010, n = 7542; 2011, n = 7252; 2012, n = 6801).

**Fig. 1:**
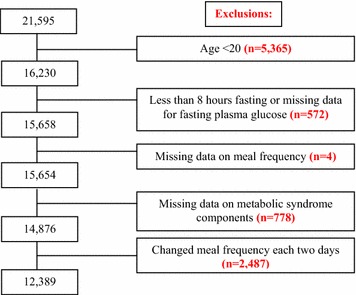
Selection of study sample from total number of participants

We excluded subjects younger than 19 years of age (n = 5365), and those who fasted less than 8 h and missed fasting plasma glucose level (n = 572). Of the remaining 15,658 subjects, we further excluded 2487 participants who reported variable meal frequency. Answers to meal frequency questions were missing in a small number of subjects (n = 4). Those with incomplete data reporting MetS components (n = 778) were also excluded. Ultimately, 12,389 participants were included in this analysis (Fig. 1). The study was approved by the Institutional Review Board of the Korea Centers for Disease Control and Prevention (KCDC), and written consent was obtained from all participants [16].

### Meal frequency and dietary assessment

A ‘meal’ was defined as one of the main eating occasions of the day, nominally occurring in the morning (‘breakfast’), at mid-day (‘lunch’), and in the evening (‘dinner’). Meal frequency was determined based on the dichotomous response (yes/no) to the question ‘Did you eat breakfast/lunch/dinner yesterday and the day before yesterday?’. We only selected patients who reported the same meal frequency for both 2 days. For example, we excluded individuals who ate three meals one day and two meals on the other day. Nutritional information was collected using a 24-h dietary recall and food questionnaire, and was administered by well-trained dietary interviewers from the National Health Industry Development Institute. Daily energy and nutrient intake, including intake of total calories (kcal/day), carbohydrates (g/day), protein (g/day) and fat (g/day), were assessed. Total energy intake was calculated based on the individual 24-h dietary recall method considering all foods including snacks which consumed during one day in the previous day. This method was validated in Korea and published by the Rural Department Administration Korean Ministry of Food, Agriculture, Forestry, and Fisheries. For analyses, individuals were categorized as eating: 3 meals/day (MF3) or 2 or fewer meals/day (MF ≤ 2). The number of eating occasions was limited to only two categories due to the small number of people who were eating 2 or fewer meals/day.

### The definition of MetS and other covariates

The presence of MetS was defined using a revised version of the ATP III criteria [17]. We defined subjects with MetS as those who met three or more of the following criteria: (1) high blood pressure (BP), ≥ 130/85 mmHg or use of anti-hypertensive agents; (2) hypertriglyceridemia, ≥ 150 mg/dL (1.693 mmol/L); (3) low HDL-C, < 40 mg/dL (1.034 mmol/L) in men and < 50 mg/dL (1.293 mmol/L) in women; (4) high fasting glucose, ≥ 100 mg/dL (5.6 mmol/L); (5) waist circumference (WC) > 90 cm in men or > 80 cm in women. Trained medical staff measured height and weight to the nearest 0.1 cm and 0.1 kg, respectively, following standardized procedures in mobile examination centers. Body mass index (BMI) was calculated as weight divided by height squared (kg/m^2^), and WC was measured at the midpoint between the inferior margin of the last rib and iliac crest in a horizontal plane, according to the World Health Organization guidelines.

Lifestyle and social factors were defined according to criteria of KNHANES. Smoking status was classified as never smoked or current smoker. Current smokers were defined as subjects who had smoked ≥ 100 cigarettes and were still smoking. Never smoker category contained former smokers. Alcohol consumption was regarded ‘yes’ if over one glass of alcohol per month in recent year. Regular physical activity was defined as at least 30 min/day for 5 days or more a week with moderate intensity. Moderate-intensity activities were defined as those causing a slight increase in heart rate and breathing compared with sedentary activities. Household income was classified into low, middle, and high income groups and was calculated by dividing the household monthly income by the square root of the household size [18]. Education level was grouped into elementary school graduate, middle school graduate, high school graduate, and college graduate or more.

### Statistical analysis

All the statistical analyses were performed using the Statistical Package for Social Sciences, version 17.0 (SPSS Inc., Chicago, IL, USA). All analyses were performed separately for men and women. Differences in anthropometric characteristics, biochemical metabolic parameters and nutrient intakes according to meal frequency were compared using Student’s *t* test or Chi square test as appropriate. Differences in MetS prevalence according to meal frequency were compared using the Chi square test. Multivariate logistic regression analysis stratified by gender was used to estimate the association between meal frequency and MetS. We constructed four multivariable regression models to explore potential confounders. The first model controlled for age. The second model consisted of total calorie intake to evaluate the associations between meal frequency and MetS when there was no difference in calorie intake. The third model consisted of smoking status, alcohol consumption and physical activity, while the fourth model was further adjusted for income and education level.

## Results

### General characteristics of study populations

Anthropometric, biochemical and metabolic parameters according to meal frequency in men and women are shown in Table 1. Men in the MF3 group were older (*P* < 0.001) and had a lower BMI (*P* < 0.001) and higher systolic blood pressure (SBP) (*P* = 0.0018) than men in the MF ≤ 2 group. In addition, men in the MF3 group were less likely to consume alcohol or smoke and had significantly higher serum fasting glucose (*P* < 0.001), HbA1c (*P* < 0.001), BUN (*P* < 0.001), and uric acid (*P* = 0.003) and lower total cholesterol (*P* = 0.032) and ALT (*P* = 0.025) than those in the MF ≤ 2 group.

**Table 1:** Baseline characteristics of the study population according to the meal frequency

	Male			Female		
	MF ≤ 2 (n = 784)	MF3 (n = 4387)	P	MF ≤ 2 (n = 1169)	MF3 (n = 6049)	P
Anthropometric parameters						
Age (years)	36.70 (0.45)	48.35 (0.32)	< 0.0001	39.45 (0.48)	49.76 (0.31)	< 0.0001
WC (cm)	84.88 (0.47)	84.08 (0.19)	0.1119	76.39 (0.41)	78.73 (0.21)	< 0.0001
BMI (kg/m^2^)	24.60 (0.17)	23.94 (0.06)	0.0003	22.97 (0.17)	23.43 (0.07)	0.0111
SBP (mmHg)	118.31 (0.65)	120.49 (0.31)	0.0018	111.39 (0.52)	117.02 (0.35)	< 0.0001
DBP (mmHg)	78.93 (0.52)	78.36 (0.25)	0.3175	72.22 (0.34)	73.51 (0.17)	0.0004
Economic status (income)			< 0.0001			
Low (%)	3.5 (0.59)	15.0 (0.68)		12.3 (1.19)	29.9 (0.94)	
Middle-low (%)	6.5 (0.97)	11.6 (0.61)		9.9 (1.19)	10.6 (0.52)	
Middle-high (%)	44.9 (2.15)	38.8 (0.98)		41.2 (1.99)	31.9 (0.87)	
High (%)	45.1 (2.16)	34.5 (1.04)		36.6 (1.89)	27.6 (0.99)	
Highest education level			0.0700			0.0074
≤ Elementary school (%)	29.6 (2.25)	24.7 (0.91)		27.9 (1.77)	26.1 (0.85)	
Middle school (%)	26.4 (1.90)	26.0 (0.93)		26.9 (1.65)	24.3 (0.72)	
High school (%)	20.8 (1.81)	25.6 (0.87)		26.6 (1.84)	25.2 (0.74)	
≥ College (%)	23.2 (1.91)	23.6 (0.90)		18.7 (1.49)	24.4 (0.84)	
Alcohol intake, n (%)	79.3 (1.74)	72.7 (0.86)	0.0006	50.5 (1.74)	35.6 (0.87)	< 0.0001
Current smoking, n (%)	60.5 (2.03)	37.6 (0.94)	< 0.0001	13.2 (1.44)	3.2 (0.32)	< 0.0001
Physical activity, n (%)	9.1 (1.23)	10.1 (0.61)	0.4602	7.4 (0.91)	8.7 (0.47)	0.1608
Biochemical metabolic parameters						
FPG (mg/dL)	94.73 (0.63)	99.93 (0.42)	< 0.0001	93.80 (0.76)	95.71 (0.35)	0.0220
T-cholesterol (mg/dL)	190.77 (1.62)	186.98 (0.73)	0.0318	186.39 (1.29)	190.42 (0.59)	0.0049
Triglyceride (mg/dL)	150.55 (5.67)	154.03 (2.30)	0.5660	107.63 (2.94)	114.61 (1.30)	0.0255
HDL-C (mg/dL)	49.45 (0.46)	49.47 (0.25)	0.9670	56.94 (0.43)	54.75 (0.22)	< 0.0001
LDL-C (mg/dL)	115.74 (2.30)	113.96 (1.13)	0.4896	109.79 (2.21)	114.09 (1.12)	0.0926
HbA1C (%)	5.62 (0.03)	5.85 (0.02)	< 0.0001	5.60 (0.03)	5.78 (0.02)	< 0.0001
Insulin	10.85 (0.38)	10.23 (0.17)	0.1340	10.51 (0.28)	10.38 (0.14)	0.6805
HOMA-IR	2.59 (0.12)	2.56 (0.05)	0.8071	2.47 (0.08)	2.50 (0.05)	0.7380
Nutrient intakes						
Total energy (kcal)	2306.73 (45.82)	2519.23 (19.08)	< 0.0001	1569.86 (24.17)	1782.80 (12.14)	< 0.0001
Carbohydrate (g/day)	321.21 (5.13)	388.40 (2.58)	< 0.0001	246.92 (3.77)	308.24 (1.99)	< 0.0001
Protein (g/day)	88.09 (2.53)	91.76 (0.96)	0.1662	57.52 (1.11)	63.54 (0.62)	< 0.0001
Fat (g/day)	57.09 (1.81)	52.22 (0.86)	0.0154	37.66 (1.02)	33.90 (0.48)	0.0004

Values are N, mean (SE) or N, percentage (SE) unless otherwise indicated

*MF* meal frequency, *WC* waist circumference, *BMI* body mass index, *SBP* systolic blood pressure, *DBP* diastolic blood pressure, *FPG* fasting plasma glucose, *T-cholesterol* total cholesterol, *HDL-C* high density lipoprotein-cholesterol, *LDL-C* low density lipoprotein-cholesterol, *BUN* blood urea nitrogen, *AST* aspartate aminotransferase, *ALT* alanine aminotransferase, *HOMA-IR* homeostasis model assessment-insulin resistance

In women, MF3 group were older (*P* < 0.001) and had significantly higher WC (*P* < 0.001), higher BMI (*P* = 0.011), SBP (*P* < 0.001), and DBP (*P* < 0.001). Women in MF ≤ 2 group were more likely to consume alcohol and smoke more. For men and women, education level and regular physical activity were not significantly different between the two groups.

Daily nutrient and energy intake in the MF3 group and MF ≤ 2 group are shown in Table 1. In men, total energy and carbohydrate intakes were significantly higher in the MF3 group than in the MF ≤ 2 group (*P* < 0.001, respectively). On the other hand, in women, total energy, carbohydrate, and protein intake were significantly higher in the MF3 group than in the MF ≤ 2 group (*P* < 0.001). In both men and women, fat intake was significantly higher in the MF ≤ 2 group than the MF3 group (*P* = 0.015, *P* = 0.0004). Although women consumed fewer calories, they consumed higher proportions of carbohydrates than men in both groups (MF3 group; 69% vs. 62%, MF ≤ 2 group; 63% vs. 56%, respectively).

In addition, we compared the data of a total 12,389 adults older than 19 years of age (5171 men, 7218 women) who were included and 3841 (1358 men, 2483 women) who were excluded. Those in the included group were older (46.7 years vs. 43.6 years) than those who were excluded. Furthermore, included subjects showed higher BP, fasting glucose (97 vs. 96 mg/dL) and HbA1c (5.7% vs. 5.6%), and lower HDL-C (49.5 vs. 50.5 mg/dL). Total energy, carbohydrate, and protein intake were significantly higher in included subjects than excluded subjects. The prevalence of MetS was higher in included subjects than excluded subjects (27% vs. 22%, *P* < 0.001). On the other hand, BMI, total cholesterol, LDL-C, and triglyceride were not significantly different between two groups. In the aspect of lifestyle factors, there was no significant difference in alcohol intake, but excluded subjects were more likely to smoke and have less regular physical activity.

### Prevalence of MetS according to meal frequency

**Fig. 2:**
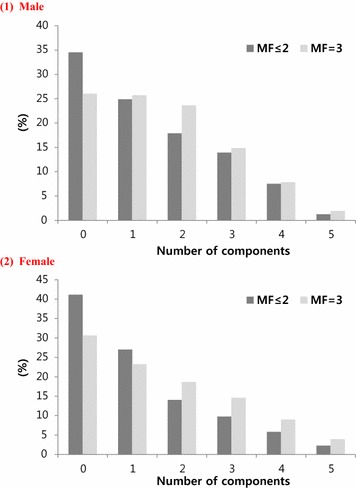
Prevalence of the number of metabolic syndrome components in subjects who eat three meals per a day (MF3) and those who eat 2 or fewer meals/day (MF ≤ 2)

Prevalence of overall MetS and each component of MetS are presented in Table 2. The prevalence of MetS was higher in women in the MF3 group than women in the MF ≤ 2 group (27.5% vs. 17.8%, *P* < 0.001). However, the prevalence of MetS in men did not differ between the MF3 group and the MF ≤ 2 group (24.6% vs. 22.7%, *P* = 0.281). In men, central obesity was more common in the MF ≤ 2 group than the MF3 group (29.3% vs. 24%), whereas high BP and high fasting plasma glucose were significantly more common in the MF3 group than the MF ≤ 2 group (44.1% vs. 32.8%, 34.1% vs. 24%, respectively). In women, all components of MetS were significantly more common in the MF3 group than the MF ≤ 2 group, although borderline significant for the high fasting plasma glucose. Prevalence of the number of MS components in MF3 group and MF ≤ 2 group are shown in Fig. 2. In both men and women of MF3 group, decreasing prevalence of the number of MS components was observed (men 26.0, 25.7, 23.6, 14.8, 7.9 and 2.0%; women 30.6, 23.2, 18.7, 14.6, 9.0 and 3.9%, respectively). MF ≤ 2 group also shown decreasing prevalence of the number of MS components (men; 34.5, 24.9, 17.9, 13.9, 7.5 and 1.3%; women 41.1, 27.0, 14.1, 9.8, 5.8 and 2.3%, respectively).

**Table 2:** Prevalence of components of metabolic syndrome according to the meal frequency

	Male			Female		
	MF ≤ 2	MF3	*P*	MF ≤ 2	MF3	*P*
MetS prevalence (%)	207 (22.7%)	1231 (24.6%)	0.2814	238 (17.8%)	1837 (27.5%)	< 0.001
Central obesity (%)	236 (29.3%)	1137 (24.0%)	0.0146	410 (31.7%)	2769 (43.1%)	< 0.001
High TG (%)	286 (35.3%)	1671 (36.7%)	0.4629	228 (18.4%)	1444 (22.5%)	0.0058
Low HDL-C (%)	145 (17.3%)	942 (19.8%)	0.1492	349 (29.6%)	2300 (36.5%)	0.0002
High BP (%)	301 (32.8%)	2239 (44.1%)	< .0001	288 (19.4%)	2436 (34.6%)	< 0.001
High FPG (%)	220 (24.0%)	1748 (34.1%)	< .0001	252 (19.8%)	1548 (23.0%)	0.0503

*MF* meal frequency, *MetS* metabolic syndrome, *TG* triglycerides, *HDL* high-density lipoprotein *BP* blood pressure, *FPG* fasting plasma glucose

### Associations between meal frequency and MetS after adjusting for total caloric intake and other covariates

Multiple logistic regression analysis with presence or absence of MetS as the dependent variable is shown in Table 3. Compared to men in the MF3 group (reference), men in the MF ≤ 2 group had an increased risk of MetS after adjusting for age and total calorie consumption (OR = 1.37, 95% CI = 1.13–1.66). Furthermore, after further adjustment for smoking status, alcohol consumption, physical activity, income, and education level, the MF ≤ 2 group had a higher risk of MetS than the MF3 group (OR = 1.37, 95% CI = 1.12–1.67). On the other hand, meal frequency was not associated with a risk of MS in women after adjusting for confounding factors including caloric intake.

**Table 3:** Multiple logistic regression analysis with presence or absence of metabolic syndrome as the dependent variable

	Male			Female		
		MF ≤ 2			MF ≤ 2	
	OR	(95% CI)	*P*	OR	(95% CI)	*P*
Unadjusted	0.92	0.77–1.09	0.3418	0.59	0.50–0.68	< 0.001
Model 1	1.37	1.13–1.66	0.0013	1.06	0.89–1.26	0.5162
Model 2	1.38	1.14–1.68	0.0012	1.07	0.89–1.29	0.4548
Model 3	1.37	1.12–1.67	0.0018	1.09	0.90–1.31	0.3705

ORs and 95% CIs in subjects who eat 2 or fewer meals/day (MF ≤ 2) compared to those who eat three meals per a day (MF3) were calculated using multiple logistic regression analysis

Model 1 adjusted for age and total calorie intake

Model 2 adjusted for model 2 plus smoking, alcohol and physical activity

Model 3 adjusted for model 3 plus income and education

*OR* odds ratio, *CI* confidence interval

## Discussion

In this study, we found that lower meal frequency was associated with increased MetS in Korean men, after adjusting for confounding factors including total calorie intake per day and physical activity. Most of the participants in KNHANES (84.2%) had 3 meals/day, indicating that, though diet patterns in Korea are rapidly changing, eating 3 ‘meals’/day still is the typical major eating habits in Koreans.

Previous studies established that calorie restriction extends the lifespan of laboratory animals and might have beneficial effects on chronic diseases such as cardiovascular disease, cancer, and neurodegenerative disorders in humans [12]. Recently, intermittent fasting has received much public attention. Experimental intermittent fasting resulted in overall reductions in calorie intake of up to 30%. However, in some studies, although the small amount of calorie restriction (5–10%) was performed, beneficial physiologic events were observed. This suggests that an extended fasting period itself may contribute to these benefits [15]. Stote et al. conducted to establish the effects of a reduced-meal frequency diet on health indicators in 15 healthy, normal-weight adults aged 40–50 years [19]. During the two 8-week treatment periods, subjects consumed all the calories needed for weight maintenance in either 3 meals/day or once meal day. Their study suggests that without a reduction in calorie intake, reduced frequency of meals does not afford major health benefits in humans.

Previous epidemiological studies have reported an inverse relationship between meal frequency and body weight and adiposity. Some studies showed higher meal frequency is related to lower body weight or obesity, while other studies didn’t show any association between meal frequency and markers of adiposity [20]. These inconsistent findings could be the result of the influence of additional confounding factors, such as total energy intake and physical activity [21]. MetS and dietary intake are closely related, and excessive energy intake causes obesity and results in increasing incidence of MetS. Therefore, a study evaluating the relationship between MetS and diet must account for total calorie intake. In this study, we checked whether there is an interaction between total calorie intake and meal frequency to examine whether we should adjust calorie intake as a confounding factor or analyze by range of calorie intake. We found that there was no significant interaction between calorie intake and meal frequency. Therefore, we adjusted for calorie intake as a confounding factor instead of analyzing data by range of calorie intake.

In the present study, the MF ≤ 2 group had higher fat intake and a greater prevalence of obesity than the MF3 group in both genders. There is evidence that fat intake is negatively correlated with insulin sensitivity [22]. Moreover, men in the MF ≤ 2 group exhibited a significantly higher risk of MetS compared to men in the MF3 group even after adjusting for various kinds of confounding factors. There are several possible explanations for the association between lower meal frequency and higher risk of MetS. When people eat only 1 or 2 meals/day, they are more likely to eat more calories at each meal. An abrupt increase in postprandial glucose level induces higher insulin response and eventually results in insulin resistance [23]. Furthermore, postprandial hyperglycemia and hyperlipidemia contribute to oxidative stress and chronic inflammation, providing a mechanistic link between these conditions and components of MetS [24, 25].

The present study found that a higher meal frequency was associated with lower prevalence of MetS in men but not in women. Men who ate more frequently were leaner than others, even if they had a higher total energy intake. In contrast, women who ate more frequently had a higher BMI and waist circumference as well as higher total energy intake than those who ate less frequently. In addition, the present results in men are consistent with previous studies that have reported that a higher eating frequency is associated with a better lipid profile. In a national population-based sample of young Australian adults, a higher number of eating occasions was significantly associated with reduced cardiometabolic risk factors (waist circumference, fasting glucose, fasting insulin, triglycerides, total cholesterol and LDL-cholesterol) in men only. They found no significant associations between eating frequency and adiposity in women [26]. Consistent with this result, several cross-sectional studies also reported that a higher eating frequency is associated with leanness in men but not in women [27–29]. Drummond et al. and Yannakoulia et al. also reported no association between eating frequency and body composition in women [28, 29]. In addition, Duval et al. reported that in a prospective observational study of 85 premenopausal women, the association between adiposity and eating frequency was no longer significant after adjusting for confounding factors, such as physical activity [21, 30]. However, the precise mechanisms responsible for gender-specific associations between meal frequency and MetS risk are not clear. In the present study, there was no difference in physical activity between men in the MF3 group and men in the MF ≤ 2 group. Furthermore, after adjusting for physical activity, lower meal frequency was associated with higher risk of MS in men. Whereas, in women, the increased risk of MetS in the MF3 group was attenuated after adjusting for confounding factors, including caloric intake and physical activity. Difference in dietary quality may contribute for these findings [31]. It is possible that women who ate more often were eating unhealthy snacks and under-reported these items. Several large surveys have shown that the proportion of total energy intake provided by ‘snacks’ in adults is around 15% [32, 33]. In a Korean study by Kim et al., the prevalence of subjects who ate one or fewer snacks per day was 83% (42% ate no snacks, and 41% ate one snack daily) [34]. However, total calorie intake in the KNHANES was calculated by a food intake survey using the individual 24-h diet recall method. Therefore, our study also took into account calories consumed from ‘snack’ foods. Another suggested explanation is the different fat distribution of men and women. Men are more likely to store excess fat centrally, whereas women store it peripherally. Because central obesity has been known as more metabolically harmful than peripheral fat, if subjects with a lower meal frequency are more likely to be obese and store fat, then this may explain why a lower meal frequency was associated with metabolic risk factors in men. Regarding the gender-specific associations between meal frequency and MetS in adults, the available scientific data are still limited. Therefore, further studies are needed to clarify this issue and, to find underlying mechanism that may be responsible for a gender-specific association.

The strengths of the present study are that it involved a large, nation-wide, population-based sample and also involved the collection of extensive data on potential confounders. Nevertheless, this study has several limitations. First, this was a cross-sectional study, and so could not determine causal relationships between meal frequency and MetS. Second, there was a potential recall bias because information was collected via self-reported questionnaires. In addition, although the present study attempted to overcome potential source of bias by excluding subjects reporting variable meal frequency for both 2 days, we cannot entirely eliminate the possibility that these inclusion/exclusion criteria may affect our study results. In fact, we found that there were significant differences of variables between subjects who were enrolled and excluded. We speculate that age of two groups may explain a considerable part of differences of metabolic and lifestyle profiles. Because younger people tend to have irregular meal pattern, they were more likely to be excluded in this study.

## Conclusion

In Korean men, lower meal frequency (2 or fewer meals/day) was associated with higher risk of MetS compared to higher meal frequency (3 meals/day). This inverse relationship between meal frequency and MetS remained even after adjusting for potential confounding factors including total calorie intake and physical activity. However, in Korean women, higher meal frequency was associated with increased risk of MetS compared to lower meal frequency. After adjusting for confounding factors, the difference in MetS risk in Korean women between the higher and lower meal frequency groups disappeared. Our study suggests that both caloric intake and meal frequency may be important factors in metabolic disorders, and their relative influence varies according to gender.

## References

[1] Grundy SM (2006). Metabolic syndrome: connecting and reconciling cardiovascular and diabetes worlds. J Am Coll Cardiol.

[2] Day C (2007). Metabolic syndrome, or what you will: definitions and epidemiology. Diab Vasc Dis Res..

[3] Lim S, Shin H, Song JH (2011). Increasing prevalence of metabolic syndrome in Korea: the Korean National Health and Nutrition Examination Survey for 1998–2007. Diabetes Care.

[4] Esmaillzadeh A, Kimiagar M, Mehrabi Y, Azadbakht L, Hu FB, Willett WC (2007). Dietary patterns, insulin resistance, and prevalence of the metabolic syndrome in women. Am J Clin Nutr.

[5] Lee KW, Cho MS (2014). The traditional Korean dietary pattern is associated with decreased risk of metabolic syndrome: findings from the Korean National Health and Nutrition Examination Survey, 1998–2009. J Med Food.

[6] Lutsey PL, Steffen LM, Stevens J (2008). Dietary intake and the development of the metabolic syndrome: the Atherosclerosis Risk in Communities study. Circulation.

[7] Sierra-Johnson J, Unden AL, Linestrand M (2008). Eating meals irregularly: a novel environmental risk factor for the metabolic syndrome. Obesity (Silver Spring)..

[8] Jaaskelainen A, Schwab U, Kolehmainen M, Pirkola J, Jarvelin MR, Laitinen J (2013). Associations of meal frequency and breakfast with obesity and metabolic syndrome traits in adolescents of Northern Finland Birth Cohort 1986. Nutr Metab Cardiovasc Dis..

[9] Carlson O, Martin B, Stote KS (2007). Impact of reduced meal frequency without caloric restriction on glucose regulation in healthy, normal-weight middle-aged men and women. Metabolism..

[10] Fontana L, Meyer TE, Klein S, Holloszy JO (2004). Long-term calorie restriction is highly effective in reducing the risk for atherosclerosis in humans. Proc Natl Acad Sci USA..

[11] Longo VD, Mattson MP (2014). Fasting: molecular mechanisms and clinical applications. Cell Metab.

[12] Mattson MP, Wan R (2005). Beneficial effects of intermittent fasting and caloric restriction on the cardiovascular and cerebrovascular systems. J Nutr Biochem.

[13] Varady KA, Hellerstein MK (2007). Alternate-day fasting and chronic disease prevention: a review of human and animal trials. Am J Clin Nutr.

[14] Shehab A, Abdulle A, El Issa A, Al Suwaidi J, Nagelkerke N (2012). Favorable changes in lipid profile: the effects of fasting after Ramadan. PLoS ONE.

[15] Anson RM, Guo Z, de Cabo R (2003). Intermittent fasting dissociates beneficial effects of dietary restriction on glucose metabolism and neuronal resistance to injury from calorie intake. Proc Natl Acad Sci USA..

[16] Kweon S, Kim Y, Jang MJ (2014). Data resource profile: the Korea National Health and Nutrition Examination Survey (KNHANES). Int J Epidemiol.

[17] Grundy SM, Cleeman JI, Daniels SR (2005). Diagnosis and management of the metabolic syndrome: an American Heart Association/National Heart, Lung, and Blood Institute Scientific Statement. Circulation.

[18] Park YW, Zhu S, Palaniappan L, Heshka S, Carnethon MR, Heymsfield SB (2003). The metabolic syndrome: prevalence and associated risk factor findings in the US population from the Third National Health and Nutrition Examination Survey, 1988–1994. Arch Intern Med.

[19] Stote KS, Baer DJ, Spears K (2007). A controlled trial of reduced meal frequency without caloric restriction in healthy, normal-weight, middle-aged adults. Am J Clin Nutr.

[20] Louis-Sylvestre J, Lluch A, Neant F, Blundell JE (2003). Highlighting the positive impact of increasing feeding frequency on metabolism and weight management. Forum Nutr..

[21] Duval K, Strychar I, Cyr MJ, Prudhomme D, Rabasa-Lhoret R, Doucet E (2008). Physical activity is a confounding factor of the relation between eating frequency and body composition. Am J Clin Nutr.

[22] Storlien LH, Baur LA, Kriketos AD (1996). Dietary fats and insulin action. Diabetologia.

[23] Basciano H, Federico L, Adeli K (2005). Fructose, insulin resistance, and metabolic dyslipidemia. Nutr Metab (Lond).

[24] Ceriello A, Bortolotti N, Motz E (1999). Meal-induced oxidative stress and low-density lipoprotein oxidation in diabetes: the possible role of hyperglycemia. Metabolism..

[25] Ceriello A, Motz E (2004). Is oxidative stress the pathogenic mechanism underlying insulin resistance, diabetes, and cardiovascular disease? The common soil hypothesis revisited. Arterioscler Thromb Vasc Biol.

[26] Smith KJ, Blizzard L, McNaughton SA, Gall SL, Dwyer T, Venn AJ (2012). Daily eating frequency and cardiometabolic risk factors in young Australian adults: cross-sectional analyses. Br J Nutr.

[27] Holmback I, Ericson U, Gullberg B, Wirfalt E (2010). A high eating frequency is associated with an overall healthy lifestyle in middle-aged men and women and reduced likelihood of general and central obesity in men. Br J Nutr.

[28] Yannakoulia M, Melistas L, Solomou E, Yiannakouris N (2007). Association of eating frequency with body fatness in pre- and postmenopausal women. Obesity (Silver Spring)..

[29] Drummond SE, Crombie NE, Cursiter MC, Kirk TR (1998). Evidence that eating frequency is inversely related to body weight status in male, but not female, non-obese adults reporting valid dietary intakes. Int J Obes Relat Metab Disord.

[30] Summerbell CD, Moody RC, Shanks J, Stock MJ, Geissler C (1996). Relationship between feeding pattern and body mass index in 220 free-living people in four age groups. Eur J Clin Nutr.

[31] Kennedy ET, Ohls J, Carlson S, Fleming K (1995). The healthy eating index: design and applications. J Am Diet Assoc.

[32] Gatenby SJ (1997). Eating frequency: methodological and dietary aspects. Br J Nutr.

[33] Dreon DM, Frey-Hewitt B, Ellsworth N, Williams PT, Terry RB, Wood PD (1988). Dietary fat: carbohydrate ratio and obesity in middle-aged men. Am J Clin Nutr.

[34] Kim S, Park GH, Yang JH, Chun SH, Woon HJ, Park MS (2014). Eating frequency is inversely associated with blood pressure and hypertension in Korean adults: analysis of the Third Korean National Health and Nutrition Examination Survey. Eur J Clin Nutr.

